# Supplementary figures and images for: Identification of immune related cells and crucial genes in the peripheral blood of ankylosing spondylitis by integrated bioinformatics analysis
Source: PeerJ. 2021 Sep 7;9:e12125. doi: 10.7717/peerj.12125 (PMC8432305; doi:10.7717/peerj.12125)

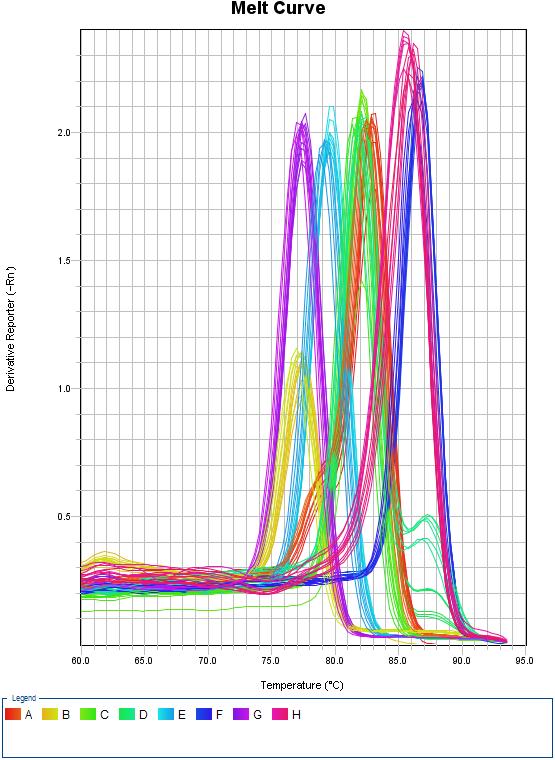

Supplement: Supplemental Information 3 [file peerj-09-12125-s003.zip › 1.0 Melt Curve.jpg]

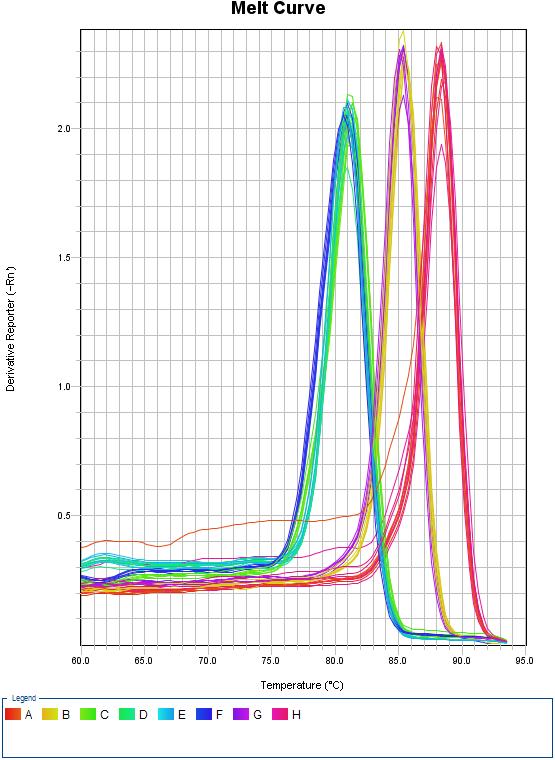

Supplement: Supplemental Information 3 [file peerj-09-12125-s003.zip › 1.1 Melt Curve.jpg]

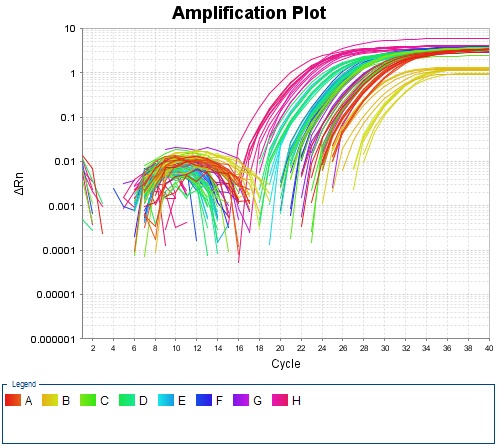

Supplement: Supplemental Information 3 [file peerj-09-12125-s003.zip › 2.0 Amplification Plot.jpg]

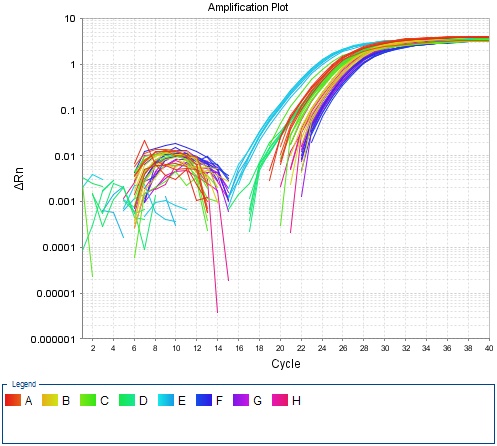

Supplement: Supplemental Information 3 [file peerj-09-12125-s003.zip › 2.1 Amplification Plot.jpg]

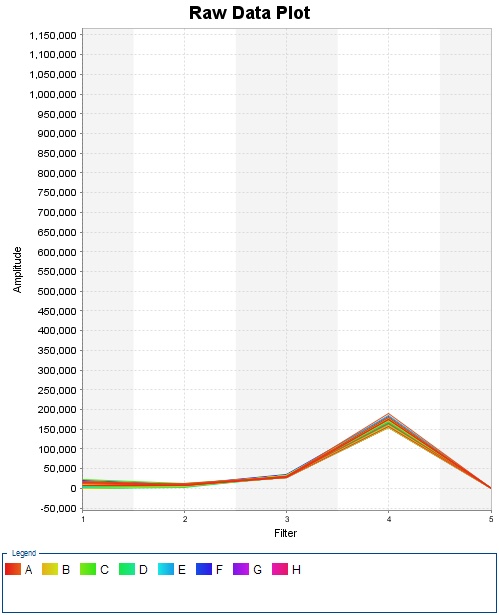

Supplement: Supplemental Information 3 [file peerj-09-12125-s003.zip › 3.0 Raw Data Plot.jpg]

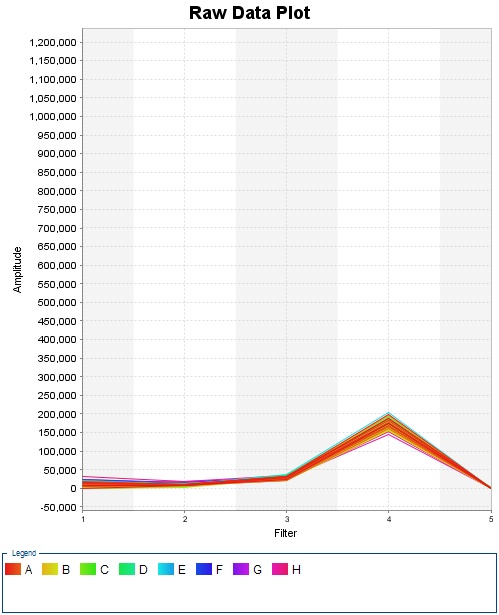

Supplement: Supplemental Information 3 [file peerj-09-12125-s003.zip › 3.1 Raw Data Plot.jpg]

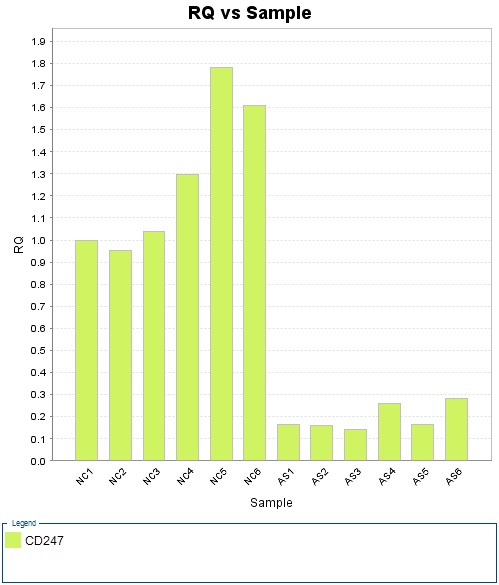

Supplement: Supplemental Information 3 [file peerj-09-12125-s003.zip › CD247.jpg]

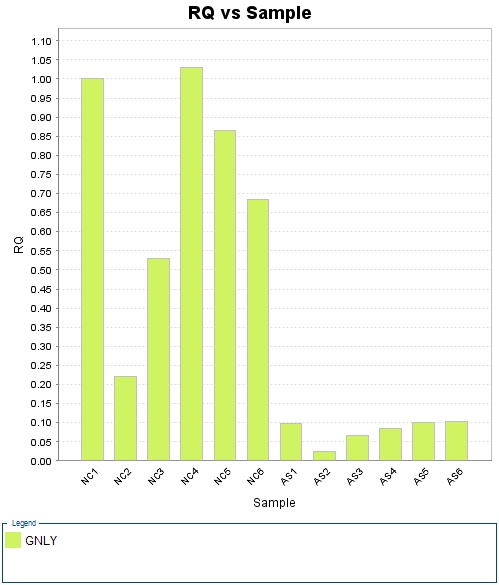

Supplement: Supplemental Information 3 [file peerj-09-12125-s003.zip › GNLY.jpg]

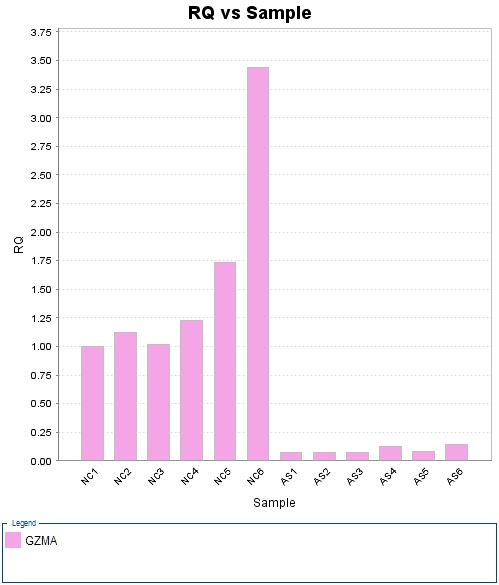

Supplement: Supplemental Information 3 [file peerj-09-12125-s003.zip › GZMA.jpg]

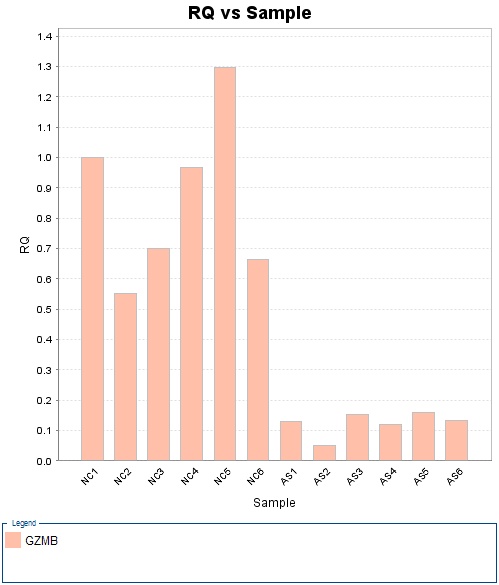

Supplement: Supplemental Information 3 [file peerj-09-12125-s003.zip › GZMB.jpg]

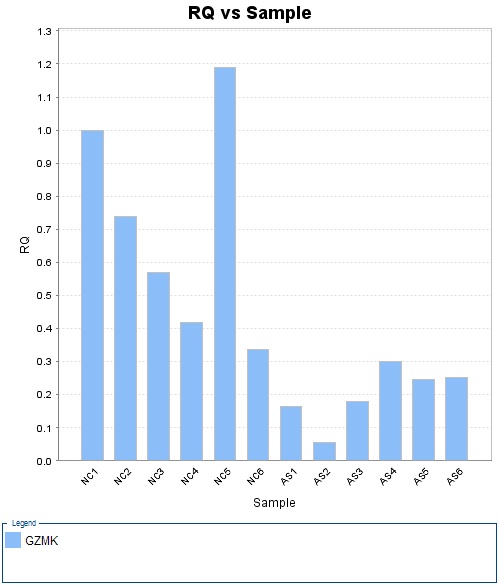

Supplement: Supplemental Information 3 [file peerj-09-12125-s003.zip › GZMK.jpg]

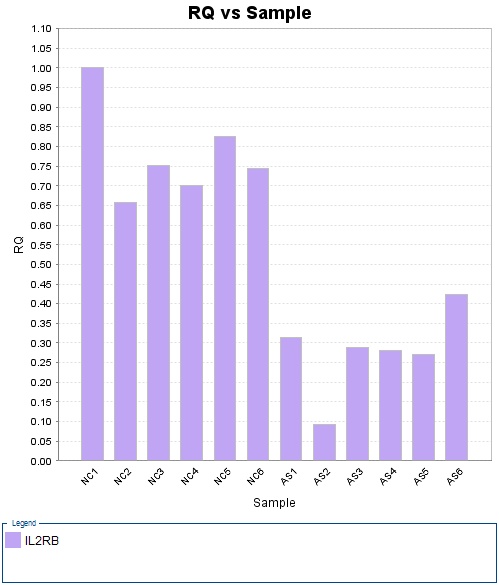

Supplement: Supplemental Information 3 [file peerj-09-12125-s003.zip › IL2RB.jpg]

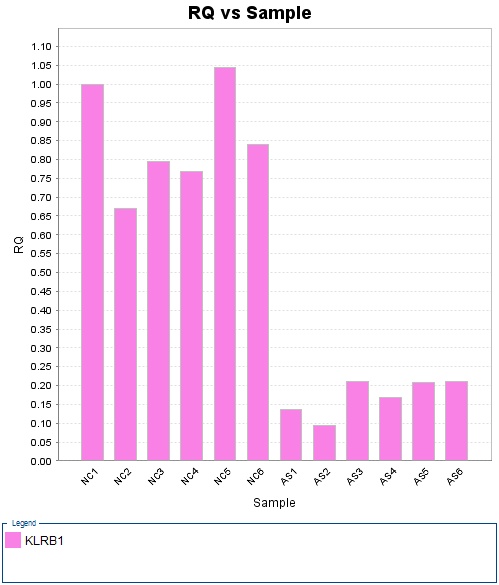

Supplement: Supplemental Information 3 [file peerj-09-12125-s003.zip › KLRB1.jpg]

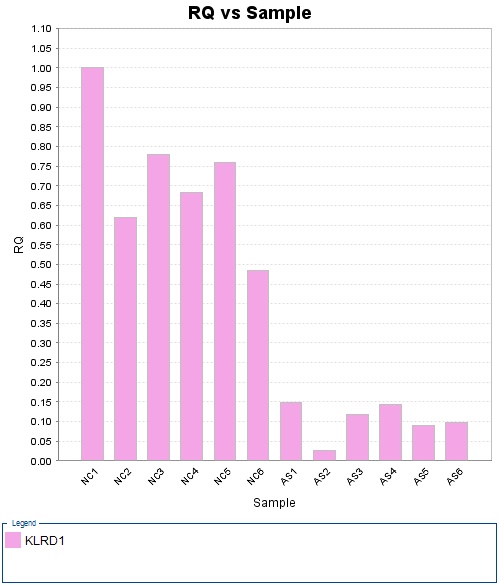

Supplement: Supplemental Information 3 [file peerj-09-12125-s003.zip › KLRD1.jpg]

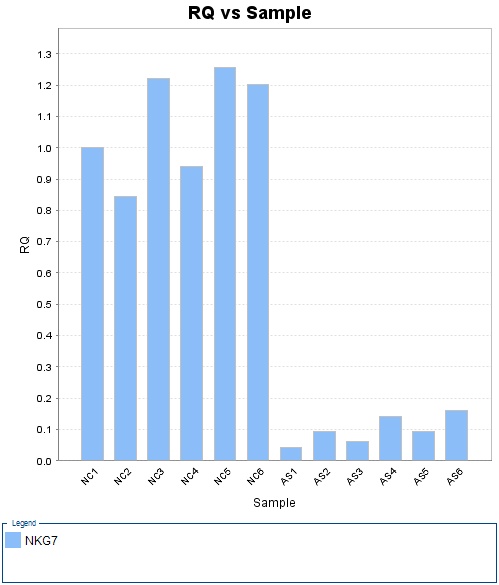

Supplement: Supplemental Information 3 [file peerj-09-12125-s003.zip › NKG7.jpg]

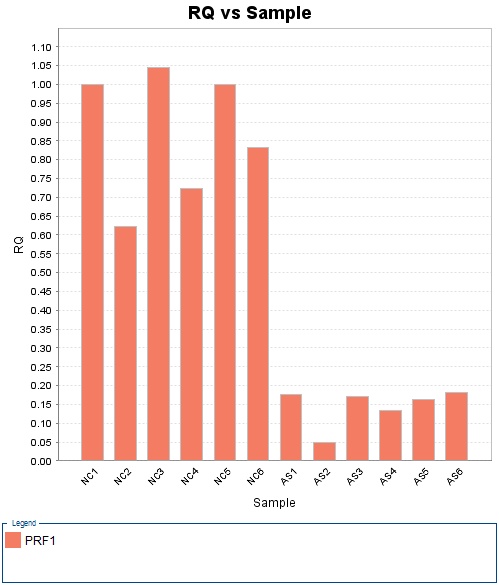

Supplement: Supplemental Information 3 [file peerj-09-12125-s003.zip › PRF1.jpg]
