# Supplementary material for: Identification of immune related cells and crucial genes in the peripheral blood of ankylosing spondylitis by integrated bioinformatics analysis
Source: PeerJ. 2021 Sep 7;9:e12125. doi: 10.7717/peerj.12125 (PMC8432305; doi:10.7717/peerj.12125)
Supplement: Supplemental Information 5 [file peerj-09-12125-s005.zip › Supplementary file 14-The baseline data of 9 AS patients.docx]

|  | AS(n=9) | | HC(n=9) |
| --- | --- | --- | --- |
| **Age** | 37.1±12.8 (19 to 62) | 39.8±13.0 (20 to 60) | |
| **Sex no. (%)** | 8M/1 F | 8 M/1 F | |
| **Disease duration (years)** | 14.9 ± 10.8 | — | |
| **HLA-B27 (%)** | 8(89) | — | |
| **ESR (0-15 mm/h)** | 20±15 (3-54) | — | |
| **CRP (0-10mg/l)** | 17.5±15.8 (3.2-52.6) | — | |
| **BASFI (0 to 100)** | 48.3±12.1(32-75) | — | |

|  | AS1 | AS2 | AS3 | AS4 | AS5 | AS6 | AS7 | AS8 | AS9 | |
| --- | --- | --- | --- | --- | --- | --- | --- | --- | --- | --- |
| **Age** | 38 | 62 | 23 | 42 | 45 | 39 | 19 | 43 | | 23 |
| **Sex** | M | M | M | M | M | F | M | M | | M |
| **Disease duration (years)** | 17 | 38 | 6 | 20 | 10 | 18 | 2 | 21 | | 2 |
| **HLA-B27 (%)** | + | + | + | - | + | + | + | + | | + |
| **ESR(0-15 mm/h)** | 54 | 36 | 9 | 10 | 14 | 15 | 25 | 3 | | 14 |
| **CRP(0-10mg/l)** | 36.1 | 12.2 | 13.6 | 4.5 | 3.2 | 20.9 | 52.6 | 3.5 | | 12.2 |
| **BASFI (0 to 100)** | 52 | 75 | 35 | 49 | 51 | 32 | 56 | 45 | | 40 |
| **Medication**  **(before treatment)** | NASIDs | NASIDs | none | none | NASIDs | none | none | NASIDs | | none |
| **Major diseases** | none | none | none | none | none | none | none | none | | none |
