# Supplementary material for: Identification of immune related cells and crucial genes in the peripheral blood of ankylosing spondylitis by integrated bioinformatics analysis
Source: PeerJ. 2021 Sep 7;9:e12125. doi: 10.7717/peerj.12125 (PMC8432305; doi:10.7717/peerj.12125)
Supplement: Supplemental Information 12 — Based on CIBERSORT algorithm, the gene expression matrix of each sample is converted into the proportion matrix of immune cells in the sample. Dark red and dark blue represent the corresponding high proportion and low proportion of immune cells respectively. [file peerj-09-12125-s012.pdf]

CIBERSORT

Input Sample	B cells naive	B cells memory	Plasma cells	T cells CD8	T cells CD4 naive	T cells CD4 memory resting	T cells CD4 memory activated	T cells follicular helper	T cells regulatory (Tregs)	T cells gamma delta	NK cells resting	NK cells activated	Monocytes	Macrophages M0	Macrophages M1	Macrophages M2	Dendritic cells resting	Dendritic cells activated	Mast cells resting	Mast cells activated	Eosinophils	Neutrophils	P-value	Pearson Correlation	RMSE
GSM616684	0.002	0.026	0	0.115	0.038	0.079	0	0	0.028	0.138	0	0.079	0.136	0.04	0	0	0	0.006	0	0	0	0.315	0.000	0.726	0.694
GSM616685	0.007	0.045	0.001	0.249	0.005	0.078	0.074	0	0	0.08	0.047	0.035	0.139	0.004	0	0	0	0.004	0	0.008	0	0.224	0.000	0.726	0.700
GSM616686	0	0.041	0	0.212	0.08	0	0.06	0	0.027	0	0.123	0.008	0.145	0.017	0	0	0	0.003	0	0.053	0	0.229	0.000	0.716	0.714
GSM616687	0	0.082	0	0.172	0.114	0.008	0.026	0	0.051	0	0.11	0	0.184	0.025	0	0	0	0.005	0	0.001	0	0.221	0.000	0.698	0.727
GSM616688	0.015	0.057	0	0.145	0.058	0.163	0.045	0	0.008	0.066	0.055	0.105	0.225	0	0	0	0	0.007	0	0	0	0.05	0.000	0.725	0.705
GSM616689	0.009	0.026	0	0.073	0.116	0.137	0.022	0	0	0.084	0.02	0.03	0.16	0	0	0	0	0.022	0	0.034	0	0.267	0.000	0.705	0.718
GSM616690	0	0.019	0	0.022	0.112	0.04	0.021	0	0.019	0.015	0.19	0	0.151	0.028	0	0	0	0.004	0	0.008	0	0.369	0.000	0.699	0.718
GSM616691	0.001	0.019	0	0.119	0.071	0.047	0.009	0	0.039	0	0.074	0.007	0.163	0.066	0	0	0	0.003	0	0.035	0	0.346	0.000	0.725	0.694
GSM616692	0	0.077	0	0.148	0.156	0.072	0.013	0	0.034	0.004	0.007	0.033	0.25	0.048	0	0	0	0.012	0	0.011	0	0.135	0.000	0.706	0.728
GSM616693	0	0.049	0	0.12	0.039	0.146	0.019	0	0.036	0	0.036	0.073	0.224	0.013	0	0	0	0.004	0	0	0	0.24	0.000	0.747	0.682
GSM616694	0.016	0.032	0	0.131	0.038	0.119	0.047	0	0.027	0.033	0.039	0.075	0.206	0.001	0	0	0	0.006	0	0.011	0	0.22	0.000	0.747	0.685
GSM616695	0.013	0.029	0	0.147	0.035	0.058	0	0	0.133	0	0.137	0.095	0.159	0.027	0	0	0	0	0	0	0	0.166	0.000	0.684	0.736
GSM616696	0	0.016	0	0.279	0.069	0	0.097	0	0.021	0	0.14	0.052	0.158	0.003	0	0	0	0.01	0	0.007	0	0.148	0.000	0.731	0.696
GSM616697	0	0.03	0	0.191	0.058	0.034	0.025	0	0.048	0	0.124	0.034	0.254	0.027	0	0	0	0.004	0.003	0	0	0.168	0.000	0.714	0.713
GSM616698	0	0.039	0	0.267	0.107	0	0.019	0	0.078	0	0.055	0.089	0.162	0.017	0	0	0	0.006	0.002	0	0	0.159	0.000	0.687	0.734
GSM616699	0	0.041	0	0.133	0.092	0	0.055	0	0.031	0	0.171	0	0.156	0.052	0	0	0	0.002	0	0.006	0	0.261	0.000	0.692	0.729
GSM616668	0	0.015	0	0.106	0.1	0.079	0.027	0	0	0	0.061	0.049	0.234	0.019	0	0	0	0.012	0	0.016	0	0.282	0.000	0.744	0.679
GSM616669	0.002	0.02	0	0.104	0.124	0	0.058	0	0.012	0	0.084	0.037	0.187	0.059	0	0	0	0.01	0.007	0	0	0.296	0.000	0.734	0.689
GSM616670	0	0.015	0	0.164	0.125	0	0.097	0	0	0.001	0.142	0.013	0.183	0.019	0	0	0	0.007	0	0.011	0	0.224	0.000	0.713	0.713
GSM616671	0	0.034	0	0.096	0.086	0.163	0.023	0	0.032	0	0.031	0.05	0.253	0.025	0	0	0	0.008	0	0.001	0	0.198	0.000	0.720	0.709
GSM616672	0	0.054	0	0.057	0.119	0.09	0	0	0.002	0.021	0.073	0.009	0.166	0.032	0	0	0	0.012	0	0	0	0.364	0.000	0.729	0.689
GSM616673	0.01	0.018	0	0.045	0.136	0.054	0.079	0	0	0.04	0.019	0.025	0.203	0.03	0	0	0	0.005	0.018	0	0	0.317	0.000	0.707	0.711
GSM616674	0.012	0.034	0	0.136	0.047	0.223	0.019	0	0	0	0.029	0.036	0.237	0.013	0	0	0	0.003	0	0.001	0	0.21	0.000	0.728	0.700
GSM616675	0	0.011	0	0.025	0.068	0.197	0.011	0	0.023	0	0.091	0	0.25	0.06	0	0	0	0.013	0	0	0	0.252	0.000	0.707	0.714
GSM616676	0.047	0.038	0	0.208	0.025	0.122	0.022	0	0.017	0.037	0.016	0.041	0.239	0	0	0	0	0.019	0.001	0	0	0.168	0.000	0.716	0.715
GSM616677	0	0.026	0	0.056	0.053	0.048	0.026	0	0.039	0.014	0.08	0	0.327	0.031	0	0	0	0.009	0.004	0	0	0.286	0.000	0.668	0.745
GSM616678	0	0.015	0	0.162	0	0.125	0.027	0	0	0	0.068	0.036	0.329	0.017	0	0	0	0.003	0.004	0	0	0.214	0.000	0.714	0.707
GSM616679	0	0.028	0	0.087	0.036	0.134	0.043	0	0	0.007	0.079	0	0.296	0	0	0	0	0.033	0	0.027	0	0.229	0.000	0.661	0.753
GSM616680	0.002	0.026	0	0.132	0.02	0.176	0.016	0	0	0.061	0.064	0.033	0.241	0.024	0	0	0	0.006	0	0	0	0.2	0.000	0.688	0.732
GSM616681	0	0.014	0	0.121	0.067	0	0.045	0	0	0.027	0.053	0	0.26	0.022	0	0	0	0.015	0	0.047	0	0.329	0.000	0.723	0.694
GSM616682	0	0.037	0	0.179	0.045	0.182	0.03	0	0	0.047	0.009	0.036	0.184	0.007	0	0	0	0.004	0	0	0	0.24	0.000	0.665	0.750
GSM616683	0.013	0.004	0	0.067	0.142	0	0.067	0	0.038	0	0.1	0.001	0.131	0.046	0	0	0	0.006	0	0.006	0	0.378	0.000	0.714	0.703
GSM1902182	0	0.049	0	0.069	0.099	0.112	0.015	0	0.019	0.062	0	0.053	0.228	0.035	0	0.003	0	0.007	0	0.019	0	0.229	0.000	0.743	0.688
GSM1902183	0	0.038	0	0.092	0.099	0.06	0.029	0	0.072	0.014	0.064	0.013	0.225	0.04	0	0	0	0.008	0	0.005	0	0.242	0.000	0.690	0.731
GSM1902184	0.007	0	0	0.183	0.015	0	0.039	0	0	0.073	0.121	0.077	0.226	0.023	0	0	0	0.007	0	0	0	0.23	0.000	0.704	0.714
GSM1902185	0	0.008	0	0.166	0.072	0	0.076	0	0.01	0.025	0.205	0.001	0.214	0.015	0	0	0	0.008	0	0.013	0	0.187	0.000	0.705	0.717
GSM1902186	0	0.024	0	0.113	0.015	0.123	0.061	0	0	0.005	0.11	0.055	0.257	0.055	0	0	0	0.002	0	0.029	0	0.15	0.000	0.735	0.701
GSM1902187	0	0.03	0	0.077	0.015	0.139	0.043	0	0.035	0.03	0.028	0.119	0.187	0.017	0	0.008	0	0.01	0.011	0	0	0.251	0.000	0.699	0.723
GSM1902188	0	0.026	0	0.236	0	0.021	0.074	0	0.012	0	0.264	0	0.176	0.012	0	0	0	0.003	0.016	0	0	0.161	0.000	0.723	0.699
GSM1902189	0.001	0.036	0	0.166	0.132	0.006	0.056	0	0	0.033	0.076	0.018	0.207	0.026	0	0	0	0.022	0	0	0	0.223	0.000	0.694	0.729
GSM1902190	0	0.034	0	0.306	0.014	0.008	0.044	0	0.011	0.133	0	0.174	0.154	0.011	0	0	0	0.002	0	0.003	0	0.105	0.000	0.753	0.666
GSM1902191	0.005	0.028	0	0.132	0.124	0.083	0.042	0	0.021	0.049	0.03	0.018	0.212	0.014	0	0	0	0.005	0.005	0	0	0.234	0.000	0.730	0.697
GSM1902192	0.001	0.027	0	0.059	0.089	0.11	0.033	0	0.007	0	0.066	0.037	0.253	0.014	0	0	0	0.006	0.035	0	0.009	0.254	0.000	0.544	0.841
GSM1902193	0	0.017	0	0.107	0.058	0.059	0.043	0	0	0.074	0.011	0	0.229	0.022	0	0	0	0.023	0	0.026	0	0.333	0.000	0.762	0.656
GSM1902194	0	0.022	0	0.074	0	0.126	0.007	0	0	0.02	0.057	0.016	0.221	0.059	0	0	0	0.008	0	0.05	0	0.339	0.000	0.739	0.679
GSM1902195	0	0.019	0	0.127	0.12	0.131	0.046	0	0.03	0	0.034	0.037	0.246	0.006	0	0	0	0.014	0	0.018	0	0.171	0.000	0.710	0.720
GSM1902196	0.004	0.022	0	0.15	0	0.093	0.034	0	0	0.124	0.025	0.111	0.198	0.014	0	0	0	0.008	0.002	0	0	0.218	0.000	0.751	0.674
GSM1902197	0	0.051	0.005	0.171	0.09	0.041	0.012	0	0.033	0.078	0.069	0.055	0.232	0	0	0	0	0.002	0	0.014	0	0.147	0.000	0.726	0.704
GSM1902198	0	0.036	0	0.129	0.08	0.05	0.002	0	0.055	0.039	0.021	0.111	0.2	0.016	0	0	0	0.037	0	0	0	0.225	0.000	0.708	0.717
GSM1902199	0	0.092	0	0.14	0.044	0.102	0.028	0	0.04	0	0.047	0.077	0.213	0.006	0	0	0	0.001	0	0.02	0	0.19	0.000	0.733	0.703
GSM1902200	0	0.043	0	0.243	0	0.034	0.07	0	0	0	0.026	0.073	0.262	0.001	0	0	0	0.028	0	0	0	0.22	0.000	0.723	0.703
GSM1902201	0	0.01	0	0.119	0.03	0.099	0.028	0	0.024	0.085	0.031	0.111	0.196	0	0	0	0	0.012	0	0.004	0	0.252	0.000	0.636	0.774
GSM1902130	0	0.009	0	0.245	0	0.062	0.12	0	0	0.015	0.073	0.081	0.205	0.005	0	0	0	0.005	0	0	0	0.181	0.000	0.723	0.703
GSM1902131	0	0.027	0	0.171	0.096	0.1	0.049	0	0.01	0	0.06	0	0.187	0.009	0	0	0	0.004	0.008	0	0	0.279	0.000	0.707	0.714
GSM1902132	0.015	0.032	0	0.087	0.091	0.094	0.049	0	0.014	0	0.054	0	0.289	0.001	0	0	0	0.011	0	0.009	0	0.255	0.000	0.735	0.688
GSM1902133	0.045	0.017	0	0.081	0.083	0.106	0.046	0	0	0.03	0.018	0.066	0.196	0	0	0	0	0.036	0	0.024	0	0.251	0.000	0.679	0.741
GSM1902134	0.01	0.02	0	0.154	0.069	0	0.036	0	0.026	0	0.058	0.049	0.271	0.037	0	0	0	0.013	0	0	0	0.257	0.000	0.610	0.793
GSM1902135	0	0.019	0	0.182	0.096	0.066	0.001	0	0.043	0	0.072	0.097	0.238	0.017	0	0	0	0.004	0.013	0	0	0.153	0.000	0.674	0.744
GSM1902136	0	0.063	0	0.121	0.098	0.122	0	0	0.059	0	0.017	0.061	0.292	0.008	0	0	0	0.016	0	0	0	0.143	0.000	0.760	0.680
GSM1902137	0	0.022	0	0.109	0.069	0.09	0.039	0	0.018	0.004	0.041	0	0.23	0.029	0	0	0	0.005	0	0	0	0.343	0.000	0.720	0.697
GSM1902138	0.003	0.018	0	0.042	0.097	0.126	0.034	0	0	0	0.077	0.005	0.189	0.063	0	0	0	0.005	0	0	0	0.34	0.000	0.744	0.675
GSM1902139	0.006	0.033	0	0.35	0	0.126	0.059	0	0	0.006	0.079	0.047	0.149	0.005	0	0	0	0.005	0.014	0	0	0.122	0.000	0.690	0.730
GSM1902140	0	0.01	0	0.122	0.09	0.063	0.01	0	0.044	0	0.012	0.034	0.183	0.02	0	0	0	0.027	0.021	0	0	0.363	0.000	0.762	0.656
GSM1902141	0	0.049	0	0.078	0.095	0.064	0.004	0	0.028	0	0.01	0.029	0.244	0.082	0	0	0	0.007	0.015	0	0	0.294	0.000	0.728	0.692
GSM1902142	0	0.029	0	0.076	0.105	0.108	0.036	0	0.023	0	0.048	0.079	0.206	0.021	0	0	0	0.005	0.001	0	0	0.263	0.000	0.718	0.705
GSM1902143	0	0.025	0	0.179	0.007	0.084	0.047	0	0	0	0.142	0.026	0.2	0.046	0	0	0	0.001	0.01	0	0	0.232	0.000	0.718	0.706
GSM1902144	0	0.029	0	0.117	0.113	0.028	0.073	0	0.009	0	0.044	0.005	0.321	0.026	0	0	0	0.008	0.02	0	0	0.208	0.000	0.748	0.682
GSM1902145	0	0.023	0	0.076	0.173	0.061	0.016	0	0.018	0	0.05	0.012	0.21	0.03	0	0	0	0.01	0	0.014	0	0.308	0.000	0.745	0.676
GSM1902146	0	0.013	0	0.058	0.075	0.15	0.027	0	0.014	0	0.147	0.028	0.215	0.033	0	0	0	0.001	0.024	0	0	0.216	0.000	0.737	0.693
GSM1902147	0	0.023	0	0.011	0.052	0	0.056	0	0.015	0.019	0.096	0.022	0.243	0.075	0	0	0	0.008	0	0.05	0	0.331	0.000	0.735	0.682
GSM1902148	0.009	0.023	0	0.177	0.118	0.011	0.032	0	0.009	0	0.078	0.073	0.216	0.052	0	0	0	0.009	0	0.033	0	0.161	0.000	0.703	0.726
GSM1902149	0.001	0	0	0.151	0.067	0	0.017	0	0.012	0.007	0.159	0	0.337	0.034	0	0	0	0.006	0	0	0	0.209	0.000	0.730	0.691
GSM1902150	0	0.025	0	0.047	0.094	0.121	0.013	0	0.019	0	0.064	0.015	0.193	0.06	0	0	0	0.003	0.007	0	0	0.34	0.000	0.740	0.678
GSM1902151	0.001	0.034	0	0.16	0.082	0.127	0.001	0	0.026	0.038	0.014	0.052	0.184	0.014	0	0	0	0.005	0	0.016	0	0.247	0.000	0.659	0.755
GSM1902152	0.003	0.037	0	0.079	0.249	0.05	0	0	0.036	0.005	0	0.039	0.208	0.019	0	0	0	0.026	0	0.008	0	0.243	0.000	0.714	0.710
GSM1902153	0	0.035	0	0.092	0.121	0.081	0	0	0.029	0.02	0	0.108	0.178	0.062	0	0	0	0.006	0	0.004	0	0.264	0.000	0.699	0.722
GSM1902154	0	0.029	0	0.138	0.086	0.148	0	0	0.031	0.004	0.076	0.012	0.199	0.014	0	0	0	0.006	0	0.01	0	0.246	0.000	0.669	0.747
GSM1902155	0	0.025	0.089	0.32	0.031	0.013	0.08	0	0.01	0	0.054	0.09	0.141	0.023	0	0	0	0.009	0	0.006	0	0.109	0.000	0.711	0.723
GSM1902156	0	0.041	0	0.045	0.067	0.068	0.023	0	0.034	0.091	0.012	0.011	0.202	0.013	0	0	0	0.015	0	0.003	0	0.375	0.000	0.781	0.632
GSM1902157	0	0.019	0	0.262	0.056	0	0.093	0	0	0.013	0	0.073	0.169	0.018	0	0	0	0.003	0	0.045	0	0.249	0.000	0.704	0.720
GSM1902158	0	0.036	0.02	0.05	0.075	0.182	0.014	0	0.022	0.025	0.014	0.027	0.218	0.05	0	0	0	0.006	0.003	0	0	0.259	0.000	0.729	0.697
GSM1902159	0	0.034	0	0.093	0.083	0.145	0.008	0	0.033	0.013	0.01	0.02	0.224	0.059	0	0	0	0.006	0.004	0	0	0.269	0.000	0.761	0.665
GSM1902160	0	0.009	0	0.015	0.099	0.031	0.024	0	0.017	0	0.068	0	0.14	0.035	0	0	0	0.019	0.026	0	0	0.516	0.000	0.717	0.710
GSM1902161	0.013	0.042	0	0.066	0.053	0.162	0.016	0	0.031	0.029	0.008	0.046	0.217	0.013	0	0	0	0.005	0	0	0	0.3	0.000	0.746	0.676
GSM1902162	0	0.063	0	0.047	0.055	0.072	0.027	0	0.031	0.001	0.058	0	0.261	0	0	0	0	0.013	0	0.034	0	0.338	0.000	0.785	0.631
GSM1902163	0.012	0.044	0	0.101	0.046	0.055	0.019	0	0.031	0.014	0.044	0.037	0.223	0.03	0	0	0	0.007	0	0.007	0	0.329	0.000	0.724	0.694
GSM1902164	0	0.042	0	0.062	0.021	0.036	0.031	0	0	0	0.082	0.048	0.17	0	0	0	0	0.025	0	0.051	0	0.431	0.000	0.750	0.667
GSM1902165	0	0.034	0	0.155	0.09	0.127	0.02	0	0	0	0.063	0.098	0.16	0.006	0	0	0	0.023	0	0	0	0.224	0.000	0.639	0.771
GSM1902166	0	0.058	0	0.092	0.148	0.166	0.025	0	0.034	0.005	0.024	0.053	0.16	0.036	0	0	0	0.006	0	0	0	0.194	0.000	0.711	0.719
GSM1902167	0	0.022	0	0.129	0.129	0.117	0.006	0	0	0.076	0	0.059	0.254	0.009	0	0	0	0.007	0	0.012	0	0.18	0.000	0.633	0.776
GSM1902168	0	0.023	0	0.084	0.207	0.114	0.035	0	0.006	0	0.034	0.001	0.148	0.019	0	0	0	0.005	0	0.007	0	0.317	0.000	0.663	0.750
GSM1902169	0	0.031	0.006	0.065	0.097	0.114	0.012	0	0.03	0	0.011	0.061	0.212	0.064	0	0	0	0.007	0	0.013	0	0.276	0.000	0.741	0.684
GSM1902170	0.016	0.048	0	0.113	0.08	0.109	0.006	0	0.03	0	0.015	0.055	0.179	0.08	0	0	0	0.004	0.008	0	0	0.258	0.000	0.723	0.704
GSM1902171	0.006	0	0	0.062	0.086	0.049	0.037	0	0.004	0	0.139	0	0.157	0.045	0	0	0	0.013	0.023	0	0	0.379	0.000	0.669	0.746
GSM1902172	0	0.04	0	0.138	0.134	0.062	0.001	0	0.042	0	0.02	0.03	0.205	0.066	0	0	0	0.003	0	0.011	0	0.248	0.000	0.709	0.715
GSM1902173	0.013	0.107	0	0.203	0.043	0.069	0	0	0.061	0.063	0.013	0.05	0.132	0	0	0	0	0.039	0	0.011	0	0.196	0.000	0.678	0.746
GSM1902174	0	0.011	0	0.376	0.082	0	0.117	0	0.001	0	0.083	0.059	0.113	0.019	0	0	0	0.003	0.006	0	0	0.129	0.000	0.674	0.742

Input Sample	B cells naive	B cells memory	Plasma cells	T cells CD8	T cells CD4 naive	T cells CD4 memory resting	T cells CD4 memory activated	T cells follicular helper	T cells regulatory (Tregs)	T cells gamma delta	NK cells resting	NK cells activated	Monocytes	Macrophages M0	Macrophages M1	Macrophages M2	Dendritic cells resting	Dendritic cells activated	Mast cells resting	Mast cells activated	Eosinophils	Neutrophils	P-value	Pearson Correlation	RMSE
GSM1902175	0.001	0.029	0	0.123	0.187	0.116	0	0	0.06	0	0.005	0.058	0.175	0.013	0	0	0	0.003	0	0.03	0	0.2	0.000	0.720	0.709
GSM1902176	0.013	0.044	0	0.08	0.059	0.124	0.01	0	0.06	0.007	0.081	0.083	0.215	0.017	0	0	0	0.01	0	0.007	0	0.191	0.000	0.698	0.727
GSM1902177	0.006	0.018	0	0.108	0.087	0	0.032	0	0.02	0	0.075	0	0.262	0.041	0	0	0	0.005	0	0.007	0	0.337	0.000	0.663	0.751
GSM1902178	0	0.015	0	0.08	0.082	0.152	0	0	0.047	0	0.044	0.038	0.236	0.051	0	0.003	0	0	0	0.014	0	0.239	0.000	0.715	0.709
GSM1902179	0	0.022	0	0.136	0.075	0.059	0.092	0	0	0	0.182	0.013	0.132	0.057	0	0	0	0.003	0.005	0	0	0.224	0.000	0.703	0.721
GSM1902180	0.012	0.006	0	0.049	0.115	0.071	0.002	0	0.038	0	0.042	0.011	0.252	0.012	0	0	0	0.017	0	0.059	0	0.312	0.000	0.736	0.684
GSM1902181	0	0.031	0	0.092	0.11	0.099	0.02	0	0	0.088	0.002	0	0.233	0.016	0	0	0	0.024	0.003	0	0	0.281	0.000	0.715	0.704
